# Editorial: Immunological non-responders in HIV infection

**DOI:** 10.3389/fimmu.2024.1487565

**Published:** 2024-09-24

**Authors:** Evgeniya Saidakova, Konstantin Shmagel, Scott Sieg, Peter Hunt

**Affiliations:** ^1^ Biology Department, Perm State University, Perm, Russia; ^2^ Institute of Ecology and Genetics of Microorganisms of the Ural Branch of the Russian Academy of Sciences, Perm Federal Research Center of the Ural Branch of the Russian Academy of Sciences, Perm, Russia; ^3^ Department of Medicine, Division of Infectious Diseases and HIV Medicine, Case Western Reserve University, Cleveland, OH, United States; ^4^ Division of Experimental Medicine, University of California, San Francisco, San Francisco, CA, United States

**Keywords:** HIV infection (HIV), antiretroviral therapy (ART), immunological non-responder (INR), incomplete immune recovery, immune restoration, multi omics analysis, flow cytometry, ELISA (enzyme linked immuno sorbent assay)

HIV-infection leads to severe depletion of CD4+ T-cells, increasing susceptibility to opportunistic infections and cancers, ultimately leading to death in the absence of treatment. Antiretroviral therapy (ART) suppresses HIV replication, which, in turn, contributes to the regeneration of the CD4+ T-cell pool. This dual virological and immunological response reduces morbidity and mortality among people living with HIV (PLWH).

In 1998, two years after the widespread use of ART, researchers observed that some patients experienced a decrease in HIV viral load without a corresponding increase in the CD4+ T-cell count. This phenomenon has been termed a “discordant response” or “immunological non-response”. Studies have found that 10 to 40% of HIV-positive individuals who start treatment at a low CD4+ T-cell count are immunological non-responders (INRs).

In recent years, numerous articles have been published on the issue of immunological non-response. Several of these were included in the Frontiers in Immunology Research Topic “Immunological Non-Responders in HIV-infection”. This Research Topic is unique because it brought together two distinct approaches to studying immunological non-response: 1) the classical approach, which focuses on the study of individual, interconnected cells and molecules; and 2) the omics approach, which considers numerous pathways simultaneously.

Classical methods like flow cytometry and ELISA provide a detailed analysis of specific immune system mechanisms at the cellular and molecular levels. These techniques can assess the functional characteristics of immune cells, such as their ability to proliferate and secrete cytokines. However, this hypothesis-driven approach is limited to the research question, potentially overlooking important details. Omics technologies, on the other hand, enable a discovery-based approach to study the immune system by examining many components including proteins, gene expression, and metabolic properties of cells simultaneously. The systematic approach helps identify connections between diverse and sometimes unexpected pathways. Nevertheless, omics approaches generate vast amounts of data that can be challenging to interpret and link to specific biological processes. Integrating both hypothesis-driven and discovery-based approaches to address the problem has been useful in exploring mechanisms of immunological non-response among patients who initiate ART.

Yan et al. conducted a review of the cellular and molecular abnormalities observed in the biomaterial of INRs. The review provided data on CD4+ T-lymphocytes, including their impaired production in the thymus and enhanced destruction through apoptosis, pyroptosis, and ferroptosis. It also discussed the functional status of CD4+ T-cells, focusing on their activation, exhaustion, and senescence. Furthermore, the review considered data on other immune cell types, including CD8+ T-lymphocytes, double-negative T-cells, B-lymphocytes, natural killers, monocytes/macrophages, and dendritic cells, and examined the levels of soluble pro- and anti-inflammatory markers in the blood of INRs.

Vos et al. expanded on a previous study by examining over 1,500 HIV-infected patients’ blood samples with flow cytometry and ELISA. The authors used an innovative statistical approach to analyze 355 immune cell populations and their ability to produce 12 cytokines in response to 13 stimuli. This analysis provided new insights into the activation and exhaustion of CD4+ T-cells in INRs, confirming and expanding on previous findings. Nevertheless, the study did not corroborate earlier observations regarding the activation and exhaustion of CD8+ T-lymphocytes in this population of PLWH. Overall, this study represents an advancement in our understanding of the complex immunological profile of INRs.

Espineira et al. review various omics studies on samples from INRs. The studies cover genomics, transcriptomics, proteomics, metabolomics, and glycomics. It highlights that disruptions in the cell cycle, apoptosis, senescence, mitochondrial function, glycolysis, and oxidative phosphorylation are detected across several levels of biological organization. The vast amount of data presented in the review emphasizes the need for a deeper understanding of immunity as a tightly interconnected system.

Lu et al. analyzed the blood plasma metabolites of ART-suppressed PLWH. The authors identified 938 molecules, 770 being endogenous to PLWH. They found biomarkers distinguishing PLWH from healthy subjects, as well as PLWH with or without ART-mediated viral suppression. Interestingly, only a few metabolites differed between those with effective immunological response to ART and INRs. Specific plasma metabolites were correlated with inflammation markers and cardiovascular disease risk factors. This study provides insights into the metabolic changes associated with HIV-infection and ART response, with implications for understanding disease progression and identifying potential therapeutic targets.

Overall, the studies presented in the Research Topic have advanced our understanding of immunological non-response to ART, revealing abnormalities across various immune cell populations and molecules. While no single factor has been identified as the sole driver of immunological non-response to ART, the insights suggest that this outcome may be a consequence of subtle defects or imbalances within the immune system, which become apparent when the mechanisms for CD4+ T-cell regeneration are activated. It is likely that these immune system defects contribute to impaired responses to vaccines, delayed recovery from immunosuppressive therapies and other biological aspects such as diminished healthspan in PLWH. Thus, a deeper understanding of immunological non-response could help improve clinical outcomes for individuals living with HIV and possibly support immune reconstitution in other immunodeficiency disorders.

